# Animal Detection and Classification from Camera Trap Images Using Different Mainstream Object Detection Architectures

**DOI:** 10.3390/ani12151976

**Published:** 2022-08-04

**Authors:** Mengyu Tan, Wentao Chao, Jo-Ku Cheng, Mo Zhou, Yiwen Ma, Xinyi Jiang, Jianping Ge, Lian Yu, Limin Feng

**Affiliations:** 1 Ministry of Education Key Laboratory for Biodiversity Science and Engineering, National Forestry and Grassland Administration Key Laboratory for Conservation Ecology of Northeast Tiger and Leopard National Park, Northeast Tiger and Leopard Biodiversity National Observation and Research Station, National Forestry and Grassland Administration Amur Tiger and Amur Leopard Monitoring and Research Center, College of Life Sciences, Beijing Normal University, Beijing 100875, China; 2 School of Artificial Intelligence, Beijing Normal University, Beijing 100875, China; 3 School of Mathematical Sciences, Beijing Normal University, Beijing 100875, China

**Keywords:** animal identification, camera trap, object detection, deep learning

## Abstract

**Simple Summary:**

The imagery captured by cameras provides important information for wildlife research and conservation. Deep learning technology can assist ecologists in automatically identifying and processing imagery captured from camera traps, improving research capabilities and efficiency. Currently, many general deep learning architectures have been proposed but few have evaluated their applicability for use in real camera trap scenarios. Our study constructed the Northeast Tiger and Leopard National Park wildlife dataset (NTLNP dataset) for the first time and compared the real-world application performance of three currently mainstream object detection models. We hope this study provides a reference on the applicability of the AI technique in wild real-life scenarios and truly help ecologists to conduct wildlife conservation, management, and research more effectively.

**Abstract:**

Camera traps are widely used in wildlife surveys and biodiversity monitoring. Depending on its triggering mechanism, a large number of images or videos are sometimes accumulated. Some literature has proposed the application of deep learning techniques to automatically identify wildlife in camera trap imagery, which can significantly reduce manual work and speed up analysis processes. However, there are few studies validating and comparing the applicability of different models for object detection in real field monitoring scenarios. In this study, we firstly constructed a wildlife image dataset of the Northeast Tiger and Leopard National Park (NTLNP dataset). Furthermore, we evaluated the recognition performance of three currently mainstream object detection architectures and compared the performance of training models on day and night data separately versus together. In this experiment, we selected YOLOv5 series models (anchor-based one-stage), Cascade R-CNN under feature extractor HRNet32 (anchor-based two-stage), and FCOS under feature extractors ResNet50 and ResNet101 (anchor-free one-stage). The experimental results showed that performance of the object detection models of the day-night joint training is satisfying. Specifically, the average result of our models was 0.98 *mAP* (mean average precision) in the animal image detection and 88% accuracy in the animal video classification. One-stage YOLOv5m achieved the best recognition accuracy. With the help of AI technology, ecologists can extract information from masses of imagery potentially quickly and efficiently, saving much time.

## 1. Introduction

Nature is degenerating globally at unprecedented rates, and various human-driven changes have accelerated biodiversity loss [1,2,3]. The Living Planet Report 2020 reveals that populations of mammals, birds, fish, amphibians, and reptiles have fallen by 68% over the past 50 years [4]. There is an urgent need to understand the mechanisms of biodiversity loss in the context of increasing anthropogenic disturbance [5,6]. Therefore, we have to obtain timely and exact information on the species’ distribution, richness, abundance, and community structure.

Camera trap surveys can provide valuable information for ecologists and wildlife conservation scientists on the species richness distribution [7,8], animal behavior [9], population density [10], community dynamics [11], and so forth [12,13]. As a non-invasive approach with good concealment, small interference, and 24 h of continuous work, camera traps prompt wide usage in wildlife management and biodiversity monitoring [14,15]. A camera trap will be automatically triggered to take photos or videos when animals pass by [16]. However, camera traps are also susceptible to complex environments (e.g., vegetation drifting with the wind, sunlight exposure, etc.), resulting in false triggers and sometimes producing many images or videos with no wildlife [17,18]. The collected images and videos have to be cleaned and sorted, which are enormously labor-intensive and time-consuming manual tasks. In addition, with the wide application of camera trap surveys, the size of datasets increases rapidly, and the data preprocessing obstacle brought by images with no wildlife in them becomes more and more prominent [19,20]. Cost-effective technologies are urgently needed to aid in ecological monitoring [21,22].

Deep learning, which can process big data automatically and build relational models in massive datasets, may be a crucial tool to help ecologists organize, process, and analyze ecological data more efficiently [19,23,24]. Many researchers have tried to use deep learning to automatically identify species and remove camera trap images without animals, which greatly saves time and labor costs [17,25,26]. Norouzzadeh used multitask models to automatically identify, count, and describe wildlife images with a high classification accuracy of 93.8% [27]. Schneider successfully solved the problem of outputting only one label for multi-species images by training object detectors with Faster R-CNN [28]. Object detection can identify the location and class of interest objects in an image and return all results, so it will further improve the ability of camera data processing [29]. Afterwards, some studies suggested that in complex natural environments, the detection of the location of animals first may be the basis for improving the classification ability [15]. Vecvanags evaluated the performance of RetinaNet and Faster R-CNN, which can provide technical support for effective monitoring and management of ungulates [30]. Nowadays, many object detection models have been proposed in the field of deep learning and more and more articles have focused on these applications in ecology. However, object detection is still a challenging task in camera trap surveys and few studies have compared the currently mainstream object detection models in real camera trap monitoring projects.

Meanwhile, the long-term development of deep learning in the ecological field requires large, diverse, accurately labeled, and publicly available datasets [31]. Many previous studies trained models using large datasets from open-source databases or citizen science platforms (e.g., the Snapshot Serengeti dataset, iNaturalist), which were almost always collected from specific regions [21,27,31]. There are few wildlife datasets for deep learning training in China. We need to be aware that geographic bias in ecological datasets may have implications on the practical application of the model [31]. Additionally, the composition of different species also shows a noticeable imbalance in some datasets [32]. It is challenging and costly in time and effort to label masses of imagery from some camera trap monitoring projects. Therefore, we should consider the actual situations when we apply automatic identification technologies to actual ecological protections. Additionally, ecology researchers in China urgently need high-quality wildlife datasets for deep learning to fill the gap.

The goals of our study were to build a wildlife dataset for deep learning and evaluate the applicability of object detection in real infrared camera working scenarios. We can summarize the main contents and contributions of our work as follows: (1) We constructed the first Northeast Tiger and Leopard National Park wildlife image dataset (NTLNP dataset). (2) We verified the performance of the object detection network in recognizing wild animals in a complex natural background and compared the efficiency of three mainstream detection networks in wildlife recognition: YOLOv5 (anchor-based one-stage), FCOS (anchor-free one-stage), and Cascade R-CNN (anchor-based two-stage). (3) We applied the trained model to videos recorded by the camera traps and evaluated its performance.

The remainder of the paper is organized as follows: Section 2 presents the materials and methods used in this study; Section 3 presents the experimental results; Section 4 discusses the experimental findings, shortages, and future work; and Section 5 presents the conclusions.

## 2. Materials and Methods

### 2.1. Dataset Construction

**Figure 1:**
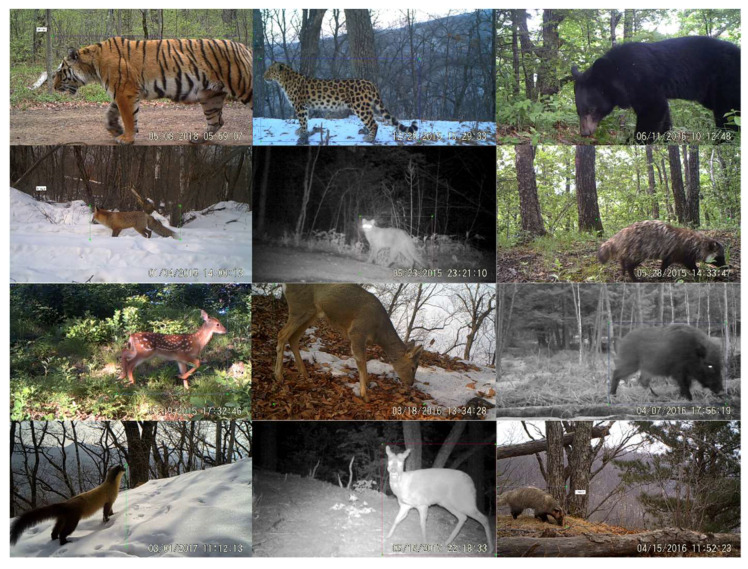
Examples of some species of the NTLNP dataset.

The data used in this study was video clips taken by infrared cameras in the Northeast Tiger and Leopard National Park from 2014 to 2020. We selected 17 main species (15 wild animals and 2 major domestic animals) as research objects, including Amur tiger (*Panthera tigris altaica*), Amur leopard (*Panthera pardus orientalis*), wild boar (*Sus scrofa*), roe deer (*Capreolus pygargus*), sika deer (*Cervus nippon*), Asian black bear (*Ursus thibetanus*), red fox (*Vulpes vulpes*), Asian badger (*Meles meles*), raccoon dog (*Nyctereutes procyonoides*), musk deer (*Moschus moschiferus*), Siberian weasel (*Mustela sibirica*), sable (*Martes zibellina*), yellow-throated marten (*Martes flavigula*), leopard cat (*Prionailurus bengalensis*), Manchurian hare (*Lepus mandshuricus*), cow, and dog. Figure 1 shows some sample images.

We used a Python script to extract images from the videos (the frame rate was 50). Limited by the number of individuals and living habits, the number of images for some species was relatively small. Except for hibernating species, images of each category included four different seasons. We carried out uniform standard manual annotation to the images. All images were labeled in Pascal VOC format using the software labelImg.

### 2.2. Object Detection Network

In the deep learning era, object detection has two main technological development routes: anchor-based and anchor-free methods while the anchor-based method includes one-stage and two-stage detection algorithms [29,33]. In the anchor-based algorithms, one-stage detection directly generates the class probability and position coordinate value of the object from the predefined anchor box; two-stage detection includes generating a region proposal from the image and generating the final target boundary from the region proposal [34]. The anchor-free method, the Keypoint-bsaed detection type such as FCOS, mainly detects target key points to produce the bounding box [35]. Therefore, the one-stage object detection algorithms may be faster, but the two-stage object detection algorithms are generally more accurate.

In this study, we applied three state-of-the-art models to identify, localize, and classify animals in a complex forest environment, namely YOLOv5, FCOS, and Cascade R-CNN [35,36]. We set up two experiment groups: one was training on day and night images jointly, and the other was training on day and night images separately.

#### 2.2.1. YOLOV5

YOLO is an acronym for ‘You only look once’. YOLOv5 is the latest generation in the YOLO series [37]. It has an anchor-based one-stage detector with a fast inference speed [38].

1. Architecture Overview

**Figure 2:**
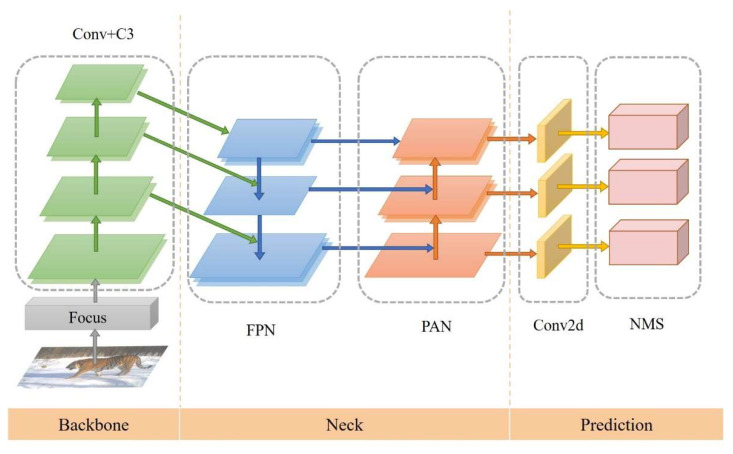
YOLOv5 structure diagram. Conv is convolution; C3 is improved from the Cross Stage Partial Network (CSP Net); Conv2d is two-dimensional convolution.

We chose three architectures: YOLOv5s, YOLOv5m, and YOLOv5l. Backbone adopts the Cross Stage Partial Network (CSPNet) [39]. Before entering the backbone network, the YOLOv5 algorithm adds the Focus module and performs downsampling by slicing the picture. The neck is in the form of a Feature Pyramid Network (FPN) plus a Path Aggregation Network (PAN) and combines three different scales of feature information [40,41]. Then, it uses the Non-Maximum Suppression (NMS) method to remove redundant prediction bounding boxes (Figure 2).

2. Implementation Details

We used the YOLOv5 framework for model training based on PyTorch [42]. The optimizer was Stochastic Gradient Descent (SGD), the momentum was set to 0.937, and the weight decay was set to 0.0005. The initial learning rate was set to 1 × 10^−2^ which would decrease linearly, the warm-up epoch was 3, and the initial warm-up momentum was 0.8. Due to the different sizes of the models, the total number of epochs and the batch size were different. The detailed settings of each model are shown in Table 1. Experiments were run on RTX A4000 GPU.

#### 2.2.2. FCOS

FCOS is a one-stage, fully convolutional object detection network that is anchor free [35]. It uses center points to replace anchor boxes for bounding box regression, which is more straightforward and flexible.

1. Architecture Overview

**Figure 3:**
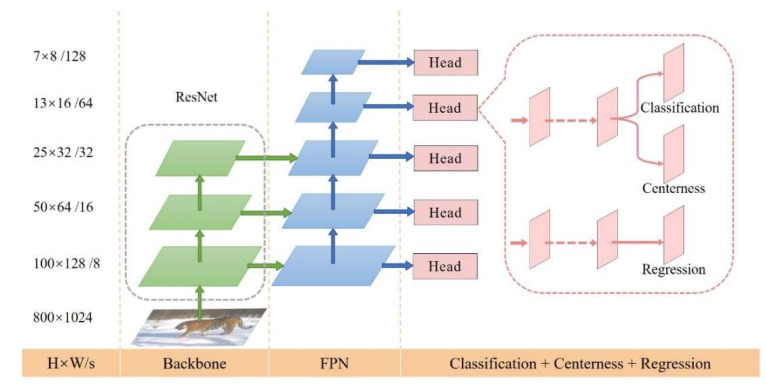
FCOS structure diagram. H × W is the height and width of feature maps. ‘/s’ (s = 8, 16, …, 128) is the downsampling ratio of the feature maps at the level to the input image [35].

The network structure consists of three main parts: backbone, FPN, and output network. The backbone network used in this experiment was ResNet50 and ResNet101 [43], which could be divided into 5 parts. It adds FPN for multi-scale feature extraction. The output network consists of Heads, each of which contains a shared part and 3 branches. Classification predicts the confidence of the existence of the target at each sampling point on the feature map, center-ness predicts the distance between the sampling point and the center of the target, and regression predicts the distance between the sampling point and the real box of the original image (Figure 3).

2. Implementation Details

We used the FCOS framework for model training based on PyTorch [35,42]. We trained 35 epochs under different backbone networks with the batch-size set to 12 and 8, respectively. In the early stage of training, the warm-up strategy was used to increase the learning rate from 0 to 2 × 10^−3^ gradually. When the training times reached 20,000 times, it reduced the learning rate to 2 × 10^−4^, and after the training times reached 27,000 times, the learning rate was reduced to 2 × 10^−5^. Experiments were run on RTX A5000 GPU.

#### 2.2.3. Cascade R-CNN

Cascade R-CNN stacks several cascade modules in the detector and uses different Intersection over Union (IoU) thresholds to train [36]. It dramatically improves the accuracy of the anchor-based two-stage object detection algorithm.

1. Architecture Overview

**Figure 4:**
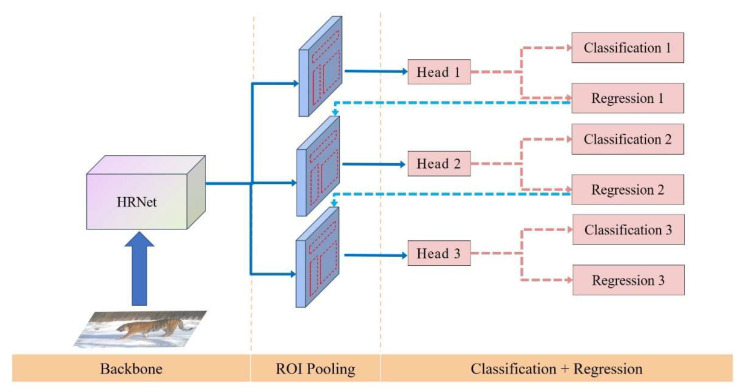
Cascade R-CNN structure diagram. ROI pooling is region-wise feature extraction [36].

We chose HRNet32 as the backbone network to perform the task of wildlife object detection in the manner of Cascade R-CNN [36,44]. HRNet achieves the purpose of strong semantic information and precise location information through parallel branches of multiple resolutions and continuous information interaction between different branches [44]. Overall, Cascade R-CNN has four stages, one Region Proposal Network (RPN) and three for detection with IoU = {0.5, 0.6, 0.7}. Sampling in the first detection stage follows Faster R-CNN [45]. In the next stage, resampling is achieved by simply using the regression output from the previous stage. The model structure is shown in Figure 4.

2. Implementation Details

We used the MMDetection framework for model training based on PyTorch [42,46]. The optimizer was Stochastic Gradient Descent (SGD), the momentum was set to 0.9, and the weight decay was set to 0.0001. The total number of epochs was 30. The learning rate was 1 × 10^−2^ and the batch size was 2. For joint training, the learning rate was 1 × 10^−2^ and the batch size was 4. In total, 500 steps were used for the warm-up. The learning rate would decrease linearly according to the epoch, and the decrease ratio was 10, in epoch 16 and epoch 19, respectively. Experiments were run on RTX 3090 GPU.

### 2.3. Evaluation Metrics

This paper used the precision, recall, and mean average precision (*mAP*) as evaluation metrics:

(1) $$Precision=\frac{TP}{TP+FP}$$

(2) $$Recall=\frac{TP}{TP+FN}$$

where true positive (*TP*) is the number of correct detections of the ground-truth bounding box, that is, the number of IoU that exceeds the threshold and is correctly classified; false positive (*FP*) is the number of incorrect detections of a nonexistent object or misplaced detections of an existing object, that is, the number of IoU not exceeding the threshold or the number of misclassification errors; and false negative (*FN*) is the number of missed detections, that is, the number of boxes that are not predicted [47]:

(3) $$AP=\int_{0}^{1}P\left(R\right)dR$$

(4) $$mAP=\frac{\sum_{i=1}^{C}AP\left(i\right)}{C}$$

*AP* (average precision) is obtained by calculating the *P-R* integral, where *P* is the precision and R is the recall. *AP* is averaged to obtain *mAP* (mean average precision), where *C* is the number of categories and in this paper, *C* = 17.

When detecting videos, we used accuracy as the evaluation metric. For a clip of the video, the final label was determined by the most frequently occurring detection results of all the frames of the target video, which were counted only if its confidence exceeded the score threshold:

(5) $$Accuracy=\frac{N}{T}$$

where *N* is the number of correctly classified videos and *T* is the total number of videos.

## 3. Results

### 3.1. NTLNP Dataset

After checking and cleaning, a total of 25,657 images were selected from 17 species categories to build the NTLNP dataset, including 15,313 images from during the day and 10,344 images from at night. The image resolution was 1280 × 720 or 1600 × 1200 pixels (Table 2). According to the ratio of 8:2, the NTLNP dataset was divided into the training set and test set. The various types of data are shown in Table 3.

### 3.2. Experimental Results

#### 3.2.1. Model Performance

Considering that the NTLNP dataset contained color images (day) and gray images (night), we investigated whether it was better when day and night images were trained separately or together. The results of each model are shown in Table 4. It was eventually discovered that the day models’ accuracy outperformed that of the night models, and when day and night images were trained jointly, all models were more accurate. Both YOLOv5 and FCOS achieved good precision and recall and Cascade_R-CNN_HRNet32 had high recall but low precision, which was 81.5%, 73.8%, and 80.9% in day, night, and day-night joint. When using *mAP* with a threshold of 0.5 IoU as the model evaluation, the average accuracy of almost all models was above 98%, and YOLOv5 had a higher value compared to the other two models. The accuracy of FCOS_Resnent50 and FCOS_Resnent101 was relatively low at night: 94.7% and 96.5%, respectively. Cascade_R-CNN_HRNet32 achieved a 97.3% accuracy in the daytime images, 97% accuracy in the nighttime images, and 98% accuracy in the day-night joint training. When using *mAP_0.5:0.95* as the metric, the models’ accuracy was between 82.4% and 88.9%.

#### 3.2.2. Species Detection and Classification

We selected YOLOv5m, FCOS_Resnet101, and Cascade_R-CNN_HRNet32, which had a better performance, to further evaluate the recognition accuracy of each species.

**Figure 5:**
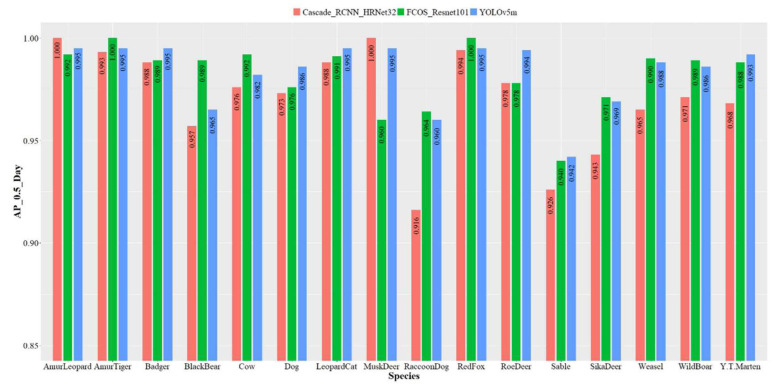
Recognition accuracy of each species of three object detection models based on the daytime dataset. The *y*-axis is the *AP* value when IOU = 0.5, ranging from 0.85–1; the *x*-axis is the species.

Since there were only 20 images of hares in the daytime, they were not considered in the model. The recognition accuracy of the 3 models trained on the daytime dataset for the 16 species is shown in Figure 5. Cascade_R-CNN_HRNet32, YOLOv5m, and FCOS_Resnet101 had a 91.6–100%, 94.2–99.5%, and 94–100% accuracy for the 16 species. Cascade_R-CNN_HRNet32 achieved a 100% recognition accuracy for Amur leopard and musk deer, and FCOS_Resnet101 for Amur tiger and red fox. The accuracy of YOLOv5m and FCOS_Resnet101 for raccoon dog reached 96% and 96.4%, respectively, which was 4.4–4.8% higher than Cascade_R-CNN_HRNet32. Sable showed the worst performance, and YOLOv5m had the relatively best accuracy of 94.2%.

**Figure 6:**
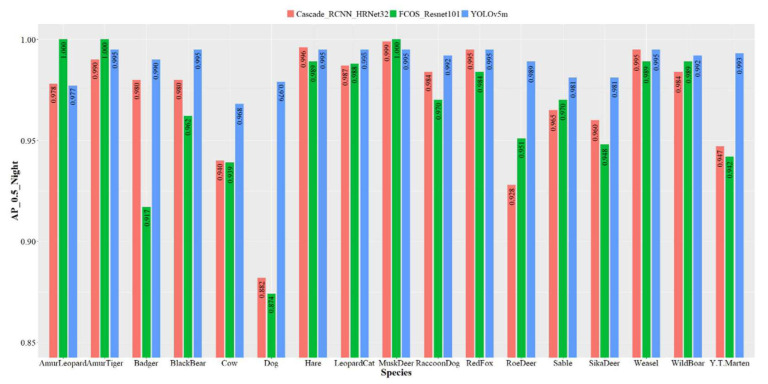
Recognition accuracy of each species of the three object detection models based on the nighttime dataset. The *y*-axis is the *AP* value when IOU = 0.5, ranging from 0.85–1; the *x*-axis is the species.

Figure 6 demonstrates the recognition accuracy of the night models. We found that the three models exhibited performance differences at night. YOLOv5m had the best accuracy in recognizing animals at night, reaching 97.7–99.5%. The accuracy of Cascade_R-CNN_HRNet32 was above 95% for most species but lower for roe deer and dogs at 92.8% and 88.2%. In contrast, FCOS_Resnet101 performed the worst at night, with significant differences among species. Amur tiger, Amur leopard, and musk deer achieved a 100% accuracy while dog and badger were only 87.4% and 91.7% accurate.

**Figure 7:**
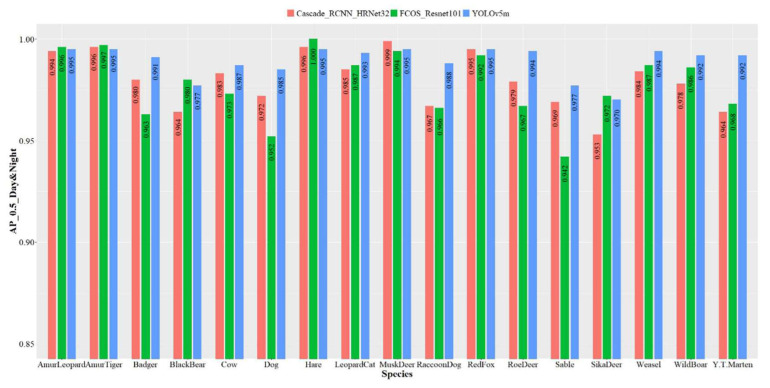
Recognition accuracy of each species of the three object detection models based on the day-night dataset. The *y*-axis is the *AP* value when IOU = 0.5, ranging from 0.85–1; the *x*-axis is the species.

Compared with separate training, the day-night jointly models achieved a better accuracy for all species (Figure 7). YOLOv5m was the best model, with an accuracy of 97–99.5%. Roe deer, badger, raccoon dog, yellow-throated marten, and dog all achieved a higher recognition accuracy than the other two models. The accuracy of FCOS_Resnet50 and Cascade_R-CNN_HRNet32 ranged from 94.2–100% and 95.3–99.9%, respectively.

**Figure 8:**
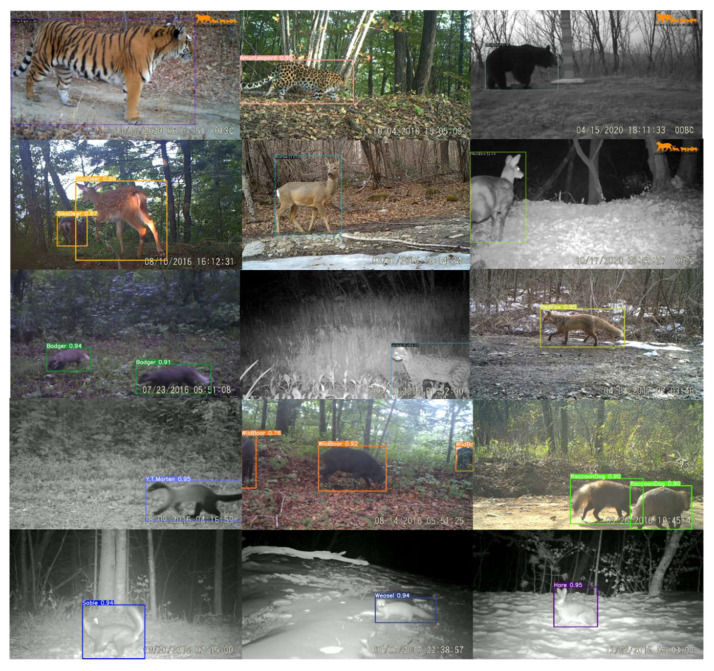
Examples of correct detection and classification.

All models had the ability to detect each object in a single image. Because different species rarely appeared in front of one camera trap at the same time, there were only images of one object or multiple objects of the same species in our dataset. Some identified images are shown in Figure 8 and more results of the different models are reported in the Supplementary Materials (Figures S1–S3).

#### 3.2.3. Video Automatic Recognition

We applied the day-night joint YOLOv5m, Cascade_R-CNN_HRNet32, and FCOS_Resnet101 to automatically recognize the videos captured by infrared cameras in the Northeast Tiger and Leopard National Park. The accuracy of the three models was tested when the score thresholds were 0.6, 0.7, and 0.8, respectively. The result is shown in Table 5. YOLOv5m showed the most robust performance among all models. When the threshold was 0.7, the accuracy was 89.6%. Cascade_R-CNN_HRNet32 was slightly inferior, obtaining the highest accuracy of 86.5% at the threshold of 0.8. The accuracy of FCOS_Resnet101 showed significant differences at different thresholds. When the threshold was 0.6, the video classification accuracy reached 91.6%. Nevertheless, when the threshold was 0.8, the recognition rate of the videos dropped sharply, eventually only reaching 64.7%.

## 4. Discussion

Open-source datasets on citizen science platforms boost interdisciplinary research, where scientists are able to train various models based on these datasets and propose optimization schemes [26,27]. However, we have to consider the geographic biases of most ecological datasets in practical applications [31]. In this study, for the first time, we constructed an image dataset of 17 species in the Northeast Tiger and Leopard National Park with standard bounding box and annotation (Table 3, NTLNP dataset). This dataset provides a great resource for exploring and evaluating the application of deep learning in the Northeast Tiger and Leopard National Park. Our dataset was small compared to large image recognition projects, but the results were relatively good and could provide a fairly effective aid in the subsequent data processing process. At the same time, the construction of the NTLNP dataset also complemented the diversity of ecological data for deep learning.

**Figure 9:**
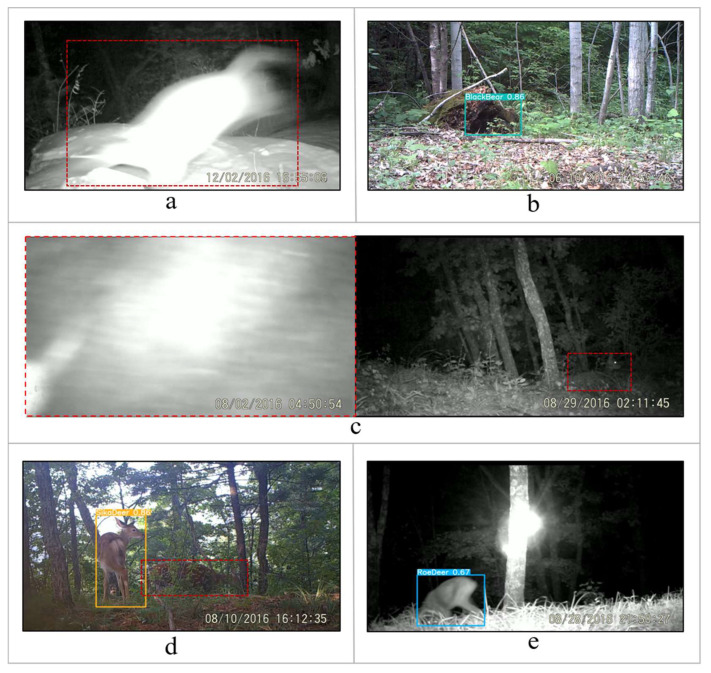
Examples of the typical failure cases of the models. (**a**) False negative or low recognition ratio due to poor image quality (blur, etc.); (**b**) Misrecognition of the background (stump, stone, fallen leave, etc.); (**c**) Inability to detect the target when animals are too close/far; (**d**) Inability to detect the target when animals are hidden or occluded; (**e**) Similar species are prone to misidentification. Red dotted boxes are added manually to show the missing targets.

By comparison, we found that day-night joint training had a better performance (Table 4), breaking our assumption that separate training would be more effective. YOLOv5, FCOS, and Cascade R-CNN all achieved high average precision: >97.9% at *mAP_0.5* and >81.2% at *mAP_0.5:0.95*, which could meet the needs of automatic wildlife recognition (Table 4). Moreover, all models exhibited similar characteristics, i.e., good performance for large targets such as Amur tiger and Amur leopard. For small targets such as badger and yellow-throated marten, the accuracy of predicting borders was reduced due to their fast movement, which would easily cause blurring in images at night (Figure 9a). Additionally, the models sometimes misidentified the background as an animal (Figure 9b). We believe that static backgrounds that closely resembled animal forms might interfere with the recognition. Additionally, when animals were too close/far or hidden/occluded, the models might have failed to detect the targets (Figure 9c,d). Some similar morphological species were prone to misidentification (Figure 9e). Overall, the recognition results were seriously affected when the image quality was poor.

In this experiment, the accuracy of the anchor-based one-stage YOLOv5 series models exceeded that of the anchor-free one-stage FCOS series models and anchor-based two-stage Cascade_R-CNN_HRNet32. Especially, YOLOv5m achieved the highest accuracy, with 98.9% for *mAP_0.5* and 88% for *mAP_0.5:0.95* (Table 4). This was inconsistent with the usual results mentioned in previous literature, where two-stage models were usually more accurate than one-stage models, and the deeper the network, the better the model performance [34]. Therefore, when applying artificial intelligence (AI), ecologists should consider the actual situation of each protected area and choose the appropriate model as a tool to help wildlife monitoring and research.

Moreover, we suggest the threshold setting of the model being tested along a suitable gradient in practical applications. When we applied the trained models to the infrared camera videos, we found that at different thresholds, the accuracy of FCOS_Resnet101 showed more significant variation while that of YOLOv5m and Cascade_R-CNN_HRNet32 was almost constant (Table 5). As can be seen, sometimes setting the threshold too high does not improve the accuracy while a problem with a low threshold is that it can lead to an increase in false positives of images without wildlife.

Finally, due to the limitations of the experimental environments, this study only compared the accuracy but failed to compare other parameters such as the running speed of the models. In follow-up studies, it is necessary to perform a comprehensive comparison before choosing the model that suits the application scenario best. In addition, we found that the background information strongly influenced the models’ performance. It should be noted that static infrared cameras are usually fixed on trees in the field, capturing large numbers of photos or videos with the same background. Beery proposed the Context R-CNN architecture, which can aggregate contextual features from other frames and leverage the long-term temporal context to improve object detection in passive monitoring [48]. The seasonal, temporal, and locational variations made the background information vary widely, so the models were prone to misjudgment for unlearned backgrounds. In the future, the selection of images of species at different times and in different geographical environments can enhance the model’s ability to learn the context. Moreover, affected by the light and geographical environments, the quality of the images and videos captured by the cameras was different, and the uncertainty of triggering, animals that were too large/small or hidden, and fast movement increases the difficulty of identification [49,50]. Attempts can be made to further improve the species recognition accuracy by combining ecological information such as the sound, activity patterns, and geographical distribution of the animals with image-based identification systems [51,52]. Furthermore, for ecological studies, distinguishing individual differences within species is also crucial, and the future incorporation of re-identification into detection systems will enable the tracking of individuals and counting of the number of species in a region [53,54,55].

## 5. Conclusions

Camera traps provide a critical aid in multifaceted surveys of wildlife worldwide while they often produce large volumes of images and videos [56]. A growing number of studies have tried to use deep learning techniques to extract effective information from massive images or videos. Our paper constructed the NTLNP dataset, which could increase the diversity of wildlife datasets, and verified the feasibility and effectiveness of object detection models for identifying wild animals in the complex forest backgrounds in the Northeast Tiger and Leopard National Park. On the NTLNP dataset, we conducted experiments on three mainstream object detection models and all models showed a satisfying performance. Moreover, we proposed that according to the deployment scenario, the dynamic selection model would achieve better results. Overall, this technology is of great practical value in helping researchers conduct more effective biodiversity monitoring, conservation, and scientific research in the Northeast Tiger and Leopard National Park.

As ecology enters the field of big data, deep learning brings a lot of hope to ecologists [19]. Although it is impossible for the model to achieve 100% accuracy, the technology will reduce the manual identification work and help ecologists quickly and efficiently extract information from massive data. In the future, in-depth interdisciplinary cooperation will further promote technological innovation in ecological research and conservation.

## Figures and Tables

**Table 1:** YOLOv5 parameter settings.

Model	Epoch	Batch Size
YOLOv5s_day	80	32
YOLOv5m_day	80	32
YOLOv5l_day	80	16
YOLOv5s_night	65	32
YOLOv5m_night	65	32
YOLOv5l_night	65	16
YOLOv5s_togather	60	32
YOLOv5m_togather	60	32
YOLOv5l_togather	45	16

**Table 2:** The main properties of the NTLNP dataset.

Species Category	No. of Total Images	No. of Daytime Images	No. of Nighttime Images	Image Resolution
17	25,657	15,313	10,344	1280 × 720/1600 × 1200

**Table 3:** NTLNP dataset and per-class training set and test set assignments.

Species	Day and Night		Day		Night	
	Training Set	Test Set	Training Set	Test Set	Training Set	Test Set
Amur tiger	1123	246	676	145	447	101
Amur leopard	1260	314	872	219	388	95
Wild boar	1801	423	1159	291	642	132
Sika dear	1726	466	1216	328	510	138
Red fox	1504	358	802	188	702	170
Raccoon dog	1169	324	248	81	921	243
Asian badger	1052	257	735	176	317	81
Asian black bear	1084	285	772	188	312	97
Leopard cat	1589	385	841	196	748	189
Roe deer	1749	374	1317	293	432	81
Siberian weasel	985	284	554	175	431	109
Yellow-throated marten	779	205	681	178	98	27
Sable	483	129	152	40	331	89
Musk deer	1045	248	216	47	829	201
Manchurian hare	1010	270	17	3	993	267
Cow	1016	284	936	263	80	21
Dog	1150	280	1056	252	94	28
Total	20,525	5132	12,250	3063	8275	2069

**Table 4:** Overall recognition accuracy of different object detection models.

Experiment	Model	Metric			
		Precision	Recall	*mAP_0.5*	*mAP_0.5:0.95*
Day&Night	YOLOv5s	0.981	0.972	0.987	0.858
	YOLOv5m	0.987	0.975	0.989	0.880
	YOLOv5l	0.984	0.975	0.989	0.878
	FCOS_Resnet50	0.969	0.892	0.979	0.812
	FCOS_Resnet101	0.963	0.882	0.978	0.820
	Cascade_R-CNN_HRNet32	0.809	0.986	0.980	0.840
Day	YOLOv5s	0.981	0.968	0.984	0.867
	YOLOv5m	0.981	0.974	0.984	0.880
	YOLOv5l	0.982	0.969	0.983	0.889
	FCOS_Resnet50	0.909	0.904	0.981	0.825
	FCOS_Resnet101	0.928	0.920	0.983	0.832
	Cascade_R-CNN_HRNet32	0.815	0.980	0.973	0.845
Night	YOLOv5s	0.956	0.972	0.984	0.850
	YOLOv5m	0.976	0.982	0.989	0.867
	YOLOv5l	0.971	0.986	0.989	0.874
	FCOS_Resnet50	0.940	0.859	0.947	0.678
	FCOS_Resnet101	0.970	0.867	0.965	0.796
	Cascade_R-CNN_HRNet32	0.738	0.981	0.970	0.824

Note: *mAP_0.5* is the average precision calculated when IoU is 0.5, *mAP_0.5:0.95* is the average precision calculated when IoU is 0.5 to 0.95 with steps of 0.05.

**Table 5:** Video classification accuracy of the three models.

Videos	Model	Acc_0.6	Acc_0.7	Acc_0.8
725	YOLOv5m	88.8%	89.6%	89.5%
	Cascade_R-CNN_HRNet32	86.3%	86.4%	86.5%
	FCOS_Resnet101	91.6%	86.6%	64.7%

Note: Acc represents *Accuracy*; Acc_0.6, 0.7, 0.8 represent the accuracy of video classification where the score threshold = {0.6, 0.7, 0.8}.

## Data Availability

NTLNP_dataset link: https://pan.bnu.edu.cn/l/s1JHuO (accessed on 1 May 2022). The key is available on request from the corresponding author.

## Supplementary Materials

The following supporting information can be downloaded at: https://www.mdpi.com/article/10.3390/ani12151976/s1, Figure S1 Examples of correct animal detection and classification usingYOLOV5m network; Figure S2 Examples of correct animal detection and classification using FCOS_Resnet101; Figure S3 Examples of correct animal detection and classification using Cascade_R-CNN_HRNet32.
