# Supplementary material for: Animal Detection and Classification from Camera Trap Images Using Different Mainstream Object Detection Architectures
Source: Animals (Basel). 2022 Aug 4;12(15):1976. doi: 10.3390/ani12151976 (PMC9367452; doi:10.3390/ani12151976)
Supplement: Supplementary file 1 [file animals-12-01976-s001.zip › animals-1795808-Supplementary.pdf]

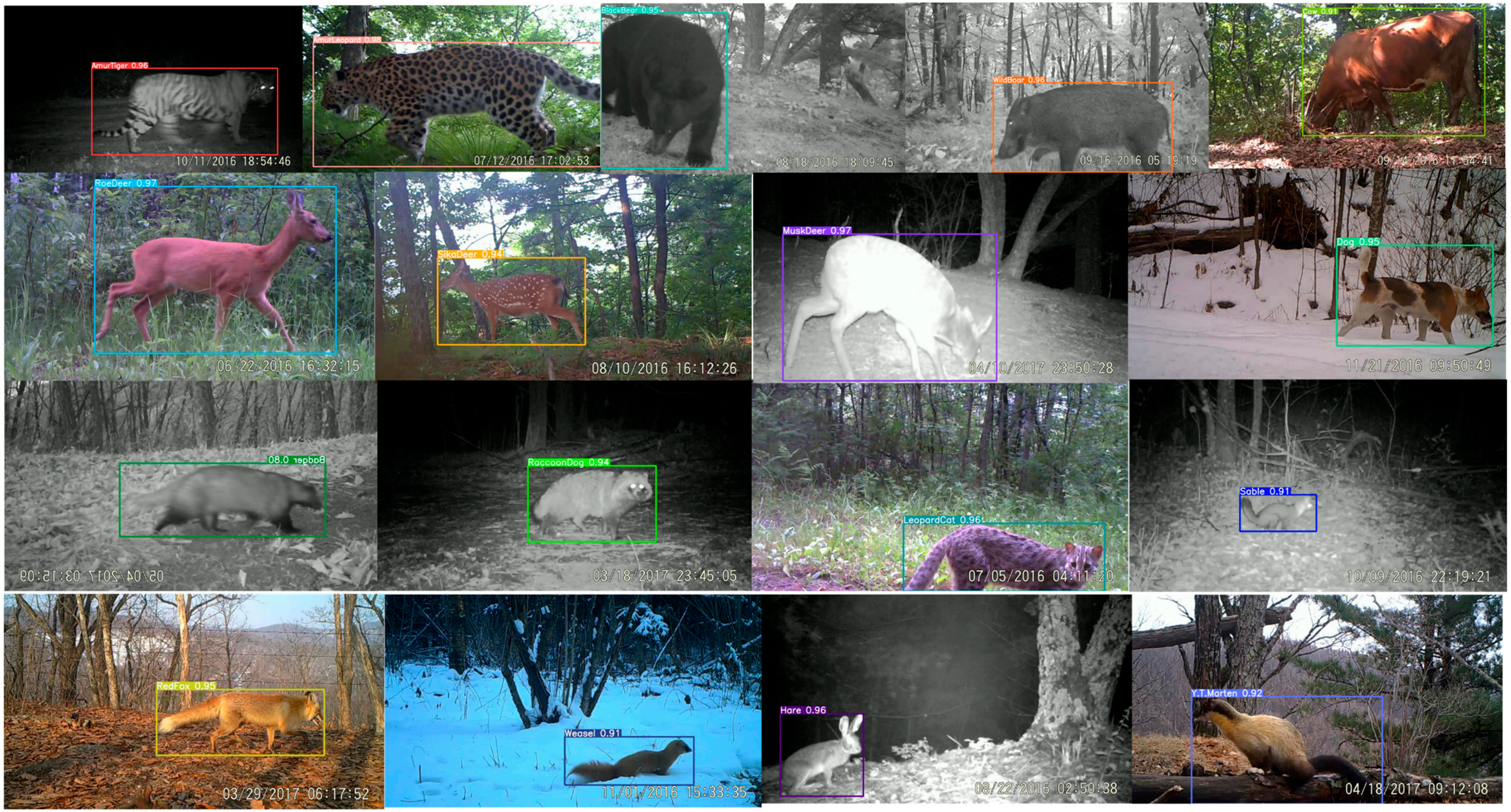

Figure S1 Examples of correct animal detection and classification usingYOLOV5m network

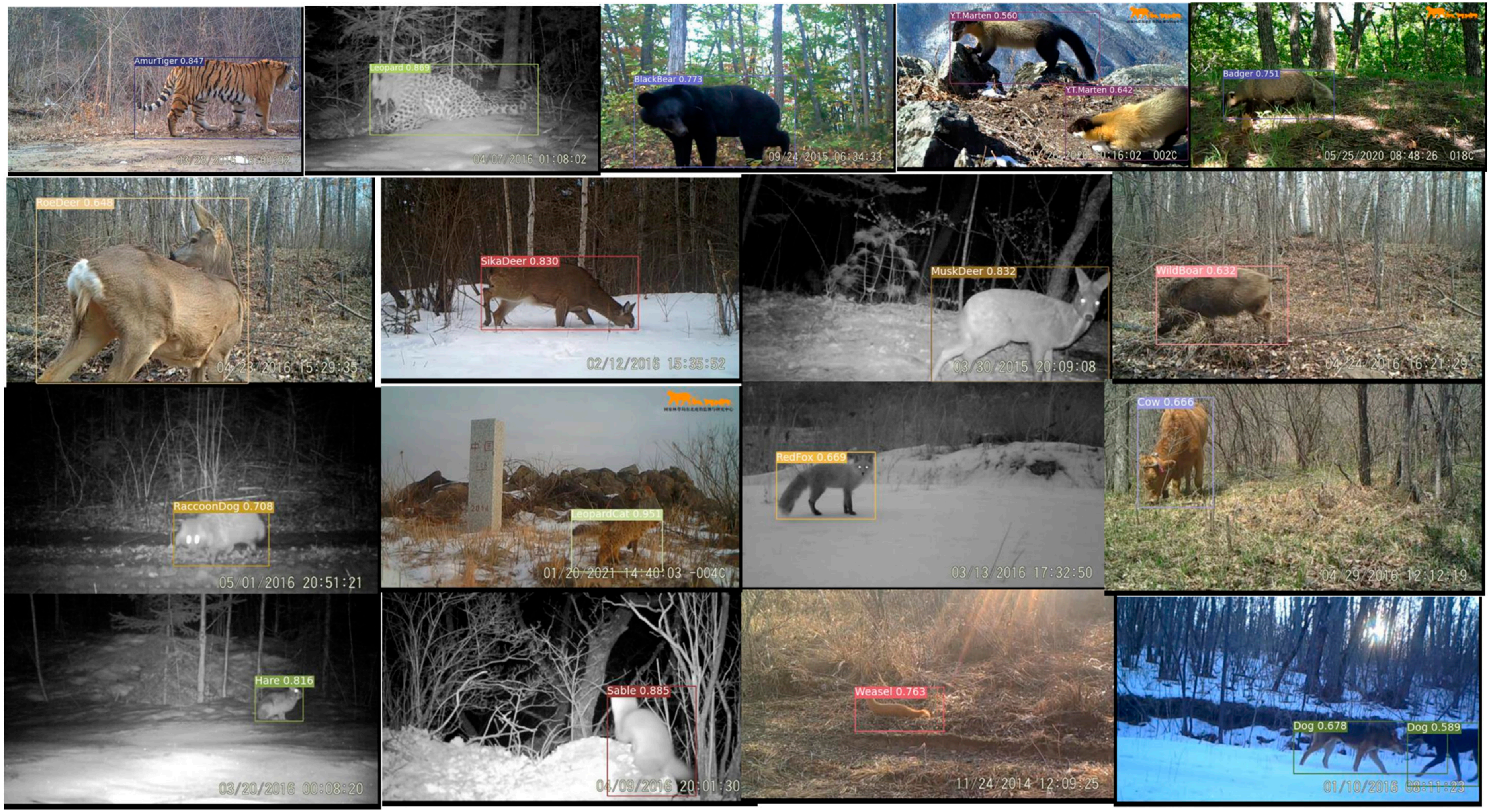

Figure S2 Examples of correct animal detection and classification using FCOS_Resnet101

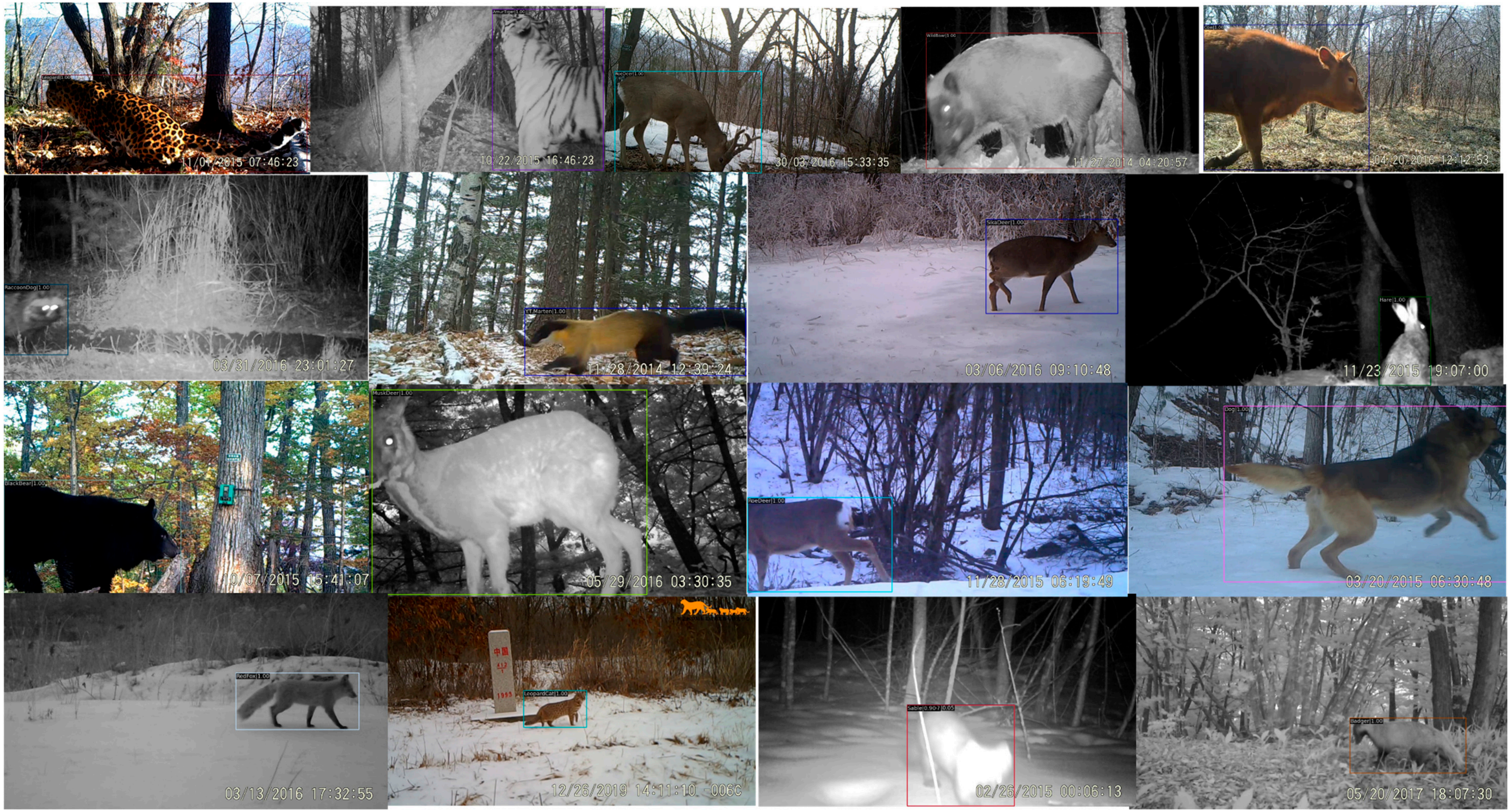

Figure S3 Examples of correct animal detection and classification using Cascade_RCNN_HRNet32
